# Heparanase 1 Upregulation Promotes Tumor Progression and Is a Predictor of Low Survival for Oral Cancer

**DOI:** 10.3389/fcell.2022.742213

**Published:** 2022-06-16

**Authors:** André A. Nimtz Rodrigues, Lucilene Lopes-Santos, Pammela A. Lacerda, Mariana F. Juste, Bruno Augusto Mariz, Débora C. Cajazeiro, Victoria Giacobbe, Rafael Borges, André Casarim, Giovanna De Sanctis Callegari, Fernando Antônio M. Claret Arcadipane, Ivan Aprahamian, Tuula Anneli Salo, Carine Ervolino De Oliveira, Ricardo D. Coletta, Taize M. Augusto, Nilva K. Cervigne

**Affiliations:** ^1^ Laboratory of Molecular Biology and Cell Culture (LBMCC), Faculty of Medicine of Jundiaí, Jundiaí, Brazil; ^2^ Department of Head and Neck Surgery, Faculty of Medicine of Jundiaí, Jundiaí, Brazil; ^3^ Department of Oral Diagnosis, School of Dentistry of Piracicaba, Campinas State University, Piracicaba, Brazil; ^4^ Independent Researcher, São Paulo, Brazil; ^5^ Institute of Pathology Cardoso de Almeida, Jundiaí, Brazil; ^6^ Department of Internal Medicine, Faculty of Medicine of Jundiaí, Jundai, Brazil; ^7^ Department of Oral and Maxillofacial Diseases, Faculty of Medicine, University of Helsinki, Helsinki, Finland; ^8^ Programa de Pós-graduação em Ciências Biológicas, Universidade Federal de Alfenas, Alfenas, Brazil; ^9^ School of Dentistry, University of Campinas, Piracicaba, Brazil; ^10^ Faculty of Medicine of Jundiaí, Jundiaí, Brazil

**Keywords:** oral squamous cell carcinoma, EMT, MEC, HPSE1, biomarker, prognosis

## Abstract

**Background:** Oral cavity cancer is still an important public health problem throughout the world. Oral squamous cell carcinomas (OSCCs) can be quite aggressive and metastatic, with a low survival rate and poor prognosis. However, this is usually related to the clinical stage and histological grade, and molecular prognostic markers for clinical practice are yet to be defined. Heparanase (HPSE1) is an endoglycosidase associated with extracellular matrix remodeling, and although involved in several malignancies, the clinical implications of HPSE1 expression in OSCCs are still unknown.

**Methods:** We sought to investigate HPSE1 expression in a series of primary OSCCs and further explore whether its overexpression plays a relevant role in OSCC tumorigenesis. mRNA and protein expression analyses were performed in OSCC tissue samples and cell lines. A loss-of-function strategy using shRNA and a gain-of-function strategy using an ORF vector targeting HPSE1 were employed to investigate the endogenous modulation of HPSE1 and its effects on proliferation, apoptosis, adhesion, epithelial–mesenchymal transition (EMT), angiogenesis, migration, and invasion of oral cancer *in vitro*.

**Results:** We demonstrated that HPSE1 is frequently upregulated in OSCC samples and cell lines and is an unfavorable prognostic indicator of disease-specific survival when combined with advanced pT stages. Moreover, abrogation of HPSE1 in OSCC cells significantly promoted apoptosis and inhibited proliferation, migration, invasion, and epithelial–mesenchymal transition by significantly decreasing the expression of N-cadherin and vimentin. Furthermore, a conditioned medium of HPSE1-downregulated cells resulted in reduced vascular endothelial growth.

**Conclusion:** Our results confirm the overexpression of HPSE1 in OSCCs, suggest that HPSE1 expression correlates with disease progression as it is associated with several important biological processes for oral tumorigenesis, and can be managed as a prognostic marker for patients with OSCC.

## Introduction

Among the most common types of cancer in the world, oral squamous cell carcinoma (OSCC) is responsible for approximately 300,000 new cases per year (Chi et al., 2015), corresponding to more than 90% of malignant tumors of oral cavity (Moro et al., 2018). In Brazil, the incidence of OSCC is considered one of the highest in the world, corresponding to the fifth most common malignancy in men and the 12th most frequent in women among all cancers in the country (Instituto Nacional de Câncer José Alencar Gomes da Silva, 2019). OSCCs are characterized by having poor prognosis and low survival, with 5-year mortality rates below 60%. In addition, the metastatic dissemination to regional lymph nodes is frequent and strongly correlated with poor disease prognosis, and it is associated with an increased risk of distant metastasis and diminished survival (Alam et al., 2011). Heparanase 1 (HPSE1) is an endo-*β*-D-glucuronidase with endoglycosidase activity capable of cleaving heparan sulfate (HS), leading to extracellular matrix (ECM) remodeling in various physiological and pathological processes and regulating the release of various HS-linked molecules, such as cytokines and enzymes involved in inflammation, wound healing, and tumor invasion (Dempsey et al., 2000; Parish et al., 2001; Jin and Zhou, 2017). The metastatic potential of tumor cells also correlates with the ability to cleave heparan sulfate proteoglycans (Vlodavsky et al., 1992; Sanderson et al., 2017). HPSE1 activity was first linked to the metastatic potential of melanoma cells in 1983 (Nakajima et al., 1983). Since then, such an association has been observed in a growing number of solid and hematological tumors, including gastric (Zheng et al., 2010), head and neck (Mogler et al., 2011), cervix (Zeng et al., 2013), pancreas (Quiros et al., 2006; Hunter et al., 2014), prostate (Zhou et al., 2008), and breast cancers (Jiao et al., 2014) (Santos et al., 2014; Xia et al., 2014; Gross-Cohen et al., 2016). The role of HPSE1 in the metastatic process appears to be associated with its ability to confer invasive properties and angiogenic potential to tumor cells (Ilan et al., 2006; Nadir et al., 2006; Vreys and David, 2007). For this reason, the use of HPSE1 inhibitors has also been strongly speculated as a potential tool for anticancer drug development (Nadir et al., 2006; Zhu et al., 2006; Pisano et al., 2014; Masola et al., 2016).

Several *in vivo* studies based on HPSE1 inhibitors support the hypothesis of the major role of HPSE1 in cancer due to its HS cleaving activity of the ECM, which contributes to tumor invasion and metastasis (Masola et al., 2018). Vlodavsky et al. (2012) reported that heparanase activity is increased in the plasma of cancer patients, and decreased heparanase expression can inhibit tumor cell metastasis. Moreover, there is evidence that the expression of genes associated with aggressive tumor behavior can be positively regulated by HPSE1 (Nadir et al., 2006; Zetser et al., 2006; Purushothaman et al., 2008; Shah et al., 2018). Thus, increased expression of heparanase is strongly correlated with metastasis, tumor vascularity, and reduced postoperative survival in cancer patients (Ilan et al., 2006; Vreys and David, 2007; Zhang et al., 2012). Several studies have shown that heparanase inhibitors (Yang et al., 2007; Ostapoff et al., 2013; Nadanaka et al., 2014) and gene silencing (Edovitsky et al., 2004; Lerner et al., 2008) can inhibit tumor growth and metastasis, providing additional evidence for the role of heparanase in tumorigenesis.

Considering the dynamics of cancer growth and the role of HPSE1 as a key in several tumor processes, it is important to combine efforts to clarify the relevance of HPSE1 in OSCCs. Our data suggest a link between the expression of HPSE1 and its potential as a prognostic marker for OSCC and shed light on our understanding of the biology for oral cancer progression, such as evidence for the action of HPSE1 in inhibiting apoptosis, promoting proliferative signaling, regulating MMP expression and EMT to facilitate invasion and establishment of the pre-metastatic niche, and increasing the bioavailability of pro-angiogenic factors. Although further investigation to delineate the crucial mechanism of action of HPSE1 in oral cavity cancer is still needed, especially to determine HPSE1-targeted therapy, our study certainly has the potential to improve patient prognosis and make therapeutic approaches more suitable for patients with oral cavity cancer.

## Materials and Methods

### Samples and Clinicopathological Data

Oral tumor tissues and non-cancerous contralateral oral mucosal tissue samples from 35 patients with OSCC were divided into two parts: one was fixed in formalin and embedded in paraffin for hematoxylin and eosin staining, as well as immunohistochemistry analysis, while the other samples were snap-frozen in liquid nitrogen and kept at −80°C until used for total RNA extraction. All these patients were diagnosed and treated at the Department of Head and Neck Surgery and Otorhinolaryngology, São Vicente de Paulo Hospital, Jundiaí, SP, Brazil. The initial diagnosis was based on clinical findings and confirmed by histopathological analysis of the specimens. A description of clinical–epidemiopathological data from all 35 OSCC patients is summarized in Supplementary Table S1. The *HPSE1* mRNA expression levels were analyzed in 31 fresh OSCC and 35 non-cancerous oral mucosal tissues by quantitative PCR (qRT-PCR). Clinicopathological features of the 31 OSCC patients, whose samples were analyzed for HPSE1 mRNA expression, are described in Table 1. Immunohistochemical analysis was also performed to confirm protein expression levels of HPSE1 in 32 of these OSCC tissue samples. After treatment, patients were regularly followed up, and the outcomes were categorized as disease-specific survival, time from treatment initiation until death due to tumor or last known date alive, time from treatment initiation until the diagnosis of the first recurrence (local, regional, or distant), or last known date alive for those without recurrence. Also, normal oral tissues were obtained from five individuals without cancer subjected to standard third molar surgery procedures. Written informed consent was obtained from each individual, and this study was approved by the Institutional Ethics Committee of the Faculty of Medicine of Jundiaí, Jundiaí, SP, Brazil (protocol number 45091715.1.0000.5412).

**TABLE 1:** Association of expression of heparanase 1 with clinicopathological parameters of tumors.

	Expression of heparanase 1		
	Low, *n* (%)	High, *n* (%)	*p* value
Age (years)			
< 62	6 (40.0)	8 (50.0)	0.58
≥ 62	9 (60.0)	8 (50.0)	
Gender			
Male	11 (73.3)	12 (75.0)	0.92
Female	4 (26.7)	4 (25.0)	
Smoking habit			
No	12 (80.0)	13 (81.2)	0.93
Yes	3 (20.0)	3 (18.8)	
Drinking habit			
No	13 (86.7)	13 (81.2)	0.68
Yes	2 (13.3)	3 (18.8)	
pT stage			
pT1 + pT2	11 (73.3)	10 (62.5)	0.52
pT3 + pT4	4 (26.7)	6 (37.5)	
pN stage			
N0	10 (66.7)	4 (25.0)	*0.02
N+	5 (33.3)	12 (75.0)	
pTNM			
Early stage (I and II)	8 (53.3)	3 (18.8)	*0.04
Advanced stage (III and IV)	7 (46.7)	13 (81.2)	
Location			
Tongue and floor of mouth	12 (80.0)	9 (56.2)	0.16
Other	3 (20.0)	7 (43.8)	
Histological grade			
Well-differentiated	10 (66.7)	4 (25.0)	*0.02
Moderately differentiated and poorly differentiated	5 (33.3)	12 (75.0)	
Treatment			
Surgery	12 (80.0)	16 (100)	0.06
Surgery + radiotherapy/chemotherapy	3 (20.0)	0	
Margin status (mm)			
≥ 5	14 (93.3)	15 (93.7)	0.96
< 5	1 (6.7)	1 (6.3)	
Perineural invasion			
No	8 (53.3)	6 (37.5)	0.38
Yes	7 (46,7)	10 (62.5)	
Lymphovascular invasion			
No	12 (80.0)	11 (68.7)	0.48
Yes	3 (20.0)	5 (31.3)	
Depth of invasion (mm)			
< 5	8 (53.3)	6 (37.5)	0.38
≥ 5	7 (46.7)	10 (62.5)	

*p < 0.05. In bold, significant association of Clinicopathological characteristics and HPSE1 overexpression in OSCC samples.

### Immunohistochemistry

Heparanase 1 (HPA, HPSE1) immunostaining was performed using the streptavidin–biotin–peroxidase complex method on 32 FFPE oral cancer specimens and five normal oral tissue specimens from non-cancerous individuals. Briefly, staining was performed using the standard method of deparaffinization and gradual rehydration, followed by heat-mediated antigen retrieval in 10 mM citric acid at pH 6.0 in a pressure cooker. The sections were further treated with 3% hydrogen peroxide to block endogenous peroxidase activity. Primary antibody incubation with polyclonal rabbit HPSE1 antibody (bs-1541R, Bioss Antibodies, United States), diluted 1:200, was carried out overnight, followed by the LSAB method (LSAB + System-HRP kit, Dako, United States). Detection was carried out using 3,3′-diaminobenzidine tetrahydrochloride (Sigma-Aldrich, United States) containing the 0.01% H_2_O_2_ detection system. Control reactions were performed by omission of the primary antibody. Immunoexpression of HPSE1 was analyzed semiquantitatively by the following criteria: the percentage of cytoplasm positivity was graded from 0 to 4 (0: no staining; 1: 1–25% staining; 3: 51–75% staining; 4: 76–100% staining) and the intensity, from 0 to 3 (0: no staining; 1: weak staining; 2: moderate staining; 3: strong staining). Tumor final scores were calculated as the sum of both categories, and the samples were classified as presenting low (0–4) or high (5–7) expression of HPSE1.

### Cell Culture

Human oral cancer cell lines SCC-4, SCC-9, SCC-15, SCC-25, and HSC-3, originally isolated from oral cancer (OSCC) of the human tongue, were obtained from the American Type Culture Collection (ATCC). The SCC-4, -9, -15, and -25 cells were grown in DMEM plus Ham’s F12 medium (DMEM/F-12), supplemented with 10% fetal bovine serum (FBS, Life Technologies, Inc.), penicillin (100 U/ml), streptomycin (100 μg/ml), and 0.4 μg/ml hydrocortisone. HSC3 human oral cancer cell line was placed in DMEM supplemented with 10% FBS, 100 units/ml penicillin, 100 μg/ml streptomycin, and 2 mM glutamine. The HPSE1-silenced (HPSE1-) and -upregulated (HPSE+) clones derived from the SCC-9 line were cultured in the same maintenance medium with the addition of 0.25 μg/ml puromycin and 5 μg/ml geneticin, respectively, for these cell expansion. Non-transformed human gingival keratinocyte cell line (HGK), obtained from mucosal keratinocytes and found to be spontaneously immortalized, was cultured in a serum-free, low-calcium medium (Gibco’s Keratinocyte-SFM; Invitrogen, United States) containing specific supplements and antibiotics, as previously described (Mäkelä et al., 1998; Rodrigues et al., 2017). Cells were maintained at 37°C in a humidified atmosphere of 5% CO_2_.

### Cell Cultures and Stable Cells Mediating Heparanase 1-Silencing and Heparanase 1 Upregulation

Considering that SCC-9 cell line had an intermediate expression level of HPSE1 within all four oral cell lines analyzed, we selected this cell line to carry out both gain- and loss-of-function *in vitro* experiments. Then, transduction of SCC-9 cells with control (scrRNA control cells) or shRNA HPSE1 sequences was performed using HuSH shRNA plasmid panel (short-hairpin RNA) following the manufacturer’s instructions (OriGene Technologies, United States/HPSE1, human, cat. No. TR307138). Briefly, after transduction, the silenced cells were selected in the presence of 1 μg/ml of puromycin (Invitrogen, United States) for 2 weeks. For the gain-of-function strategy, the transduction of SCC-9 was performed using TrueORF cDNA clones and PrecisionShuttle vector system containing the human HPSE1 cDNA clone (ORF with C-terminal GFP tag) (cat. No. RG219659) along with control SSC-9 Mock transduced cells following the manufacturer’s instructions (OriGene Technologies, United States). Then, after transduction, these cells were selected in the presence of 500 μg/ml of geneticin (ThermoFisher, United States) for 2 weeks. The insertion of the plasmids in the clones of transduced cells under- and overexpressing HPSE1 was confirmed by PCR with sequences 5′ primer and 3′ primer of the Origen Kit components before the expansion of the transduced clones.

### Real-Time Quantitative PCR

Real-time quantitative PCR was carried out to determine the efficiency of HPSE 1 silencing and upregulation in oral cancer cell lines and to investigate HPSE1 expression in oral cancer and non-cancerous oral tissue samples. Total RNA was isolated and purified using the TRIzol reagent method in accordance with the manufacturer’s protocol (Invitrogen, United States). Reverse transcription reactions were conducted with a single step with 1 ug of RNA (each sample) using the commercial kit Luna OneStep qPCR (Biolabs) in accordance with the manufacturer’s protocol. All qRT-PCRs were prepared and run in triplicates using the quantitative thermal cycler 7500 Real-Time PCR-System (Applied Biosystem, United States). Gene expressions of *HPSE1* were determined by the relative quantification using the 2^−ΔΔCt^ method, and the housekeeping gene *PPIA* (cyclophilin A) was used as a reference gene for data normalization. For the *HPSE1* expression analysis in the clinical samples, a pool of five normal oral tissues from individuals without cancer was used as the reference for the relative quantification. The amplification conditions were standardized following the manufacturer’s protocol as follows: 10 min at 55°C for enzyme activation, 1 min at 95°C for initial denaturation, followed by 45 cycles of denaturation at 95°C for 10 s, and 60 s at 60°C for pairing and extending the primers. At the end of amplification, an additional dissociation step was included (45 cycles with a decrease of 1°C every 15 s, starting at 95°C) to generate a dissociation curve (melting curve) necessary to confirm the specificity of the amplified product. Additionally, the expression analysis of markers relevant to invasion (i.e., EMT markers—E-cadherin, N-cadherin and vimentin; and ECM remodeling markers—MMP2 and MMP9) and angiogenesis (VEGFA) was carried out in cell lines and HPSE1-silenced and -upregulated clones, using qPCR as previously described (Rodrigues et al., 2017). The sequence of primers used is shown in Supplementary Table S2.

### Western Blotting Analysis

Western blotting was performed to determine the HPSE1 protein expression levels of parental OSCC cell line (WT), and the silenced and upregulated clones. The cells were washed with cold PBS and lysed with a buffer containing 50 mM NaCl, 0.5% Triton X-100, 0.5 mg EDTA/mL, and 10 μL/ml cocktail protease inhibitor (cat. P-8340—Sigma Chemical Co., St. Louis, MO, United States). After centrifugation (10,000×g at 4°C, 10 min), protein concentrations were determined using the Bradford method following the manufacturer’s instructions (Bio-Rad Protein Assay, Bio-Rad, United States). A total of 30 ug of protein was subjected to SDS-PAGE electrophoresis and transferred to nitrocellulose membranes (Bio-Rad, United States), which were blocked with non-fat dry milk (0.1% of PBS with Tween 20) and incubated overnight (4°C) with specific primary antibodies (HPSE1/1:1000, Bioss-Bs-1541R). The membranes were washed and incubated with horseradish peroxidase-conjugated secondary anti-rabbit antibody (1:2000/Cell Signaling Technology), followed by detection with enhanced chemiluminescence (GE Healthcare, United States). Anti-*β*-actin was employed as the loading control (AC-74, Sigma Aldrich, St. Louis, MO). GAPDH (1:1000/Santa Cruz Biotechnology- Sc-25778) was used as the loading control. Immunoreactive bands were detected using enhanced chemiluminescence (ECL) Western Blotting System (GE Healthcare, United States) by G-Box system (Syngene).

### Immunofluorescence Assay

To determine the localization of HPSE1, we used the immunofluorescence assay. Briefly, the parental oral cancer cell line (W)T, stable inhibition clone shRNA HPSE1, and upregulated clone orfRNA HPSE1+ were plated/well at a density of 5 × 10^4^ (24-well plate) containing sterile glass coverslips. The immunofluorescence (IF) samples were fixed in paraformaldehyde (4%), and the primary antibodies were diluted and applied to the cells (1: 250 −1 ml 3% BSA in TBS-T and 4 µL of HPA antibody −1). The secondary antibody solution (1: 1000 −1 ml of 3% BSA in TBS-T, 1 µL of Rabbit Alexa Fluor 488) was added to each cell/well, and then the cells were mounted onto glass slides with Vectashield mounting medium (Vector Labs, United States). The slides were visualized under a Leica fluorescence microscope (LEIKA, Germany) with a 40 × or ×60 × oil immersion objectives.

### Cell Cycle Analysis

The distribution of cells in the cell cycle was evaluated by flow cytometry. Cells were incubated in DMEM/F-12 without serum for 24 h and then trypsinized, resuspended in 70% ethanol, treated with 10 μg/ml of RNAse (Sigma-Aldrich, United States), and stained with 50 μg/ml of propidium iodide (Sigma-Aldrich, United States). DNA contents were then analyzed using an FACSCalibur flow cytometer (BD Biosciences, United States).

### Apoptosis Analysis

For the apoptosis assay, cells were resuspended in binding buffer (10 mM HEPES pH 7.4, 150 mM NaCl, 5 mM KCl, 1 mM MgCl_2_, and 1.8 mM CaCl_2_) containing Annexin V-Alexa Fluor 647 and 7-AAD (BD Biosciences, United States). Cell apoptosis, characterized by positivity for annexin V, was detected using an FACSCalibur flow cytometer (BD Biosciences, United States). A minimum of 10,000 events were analyzed for each sample.

### Cell Viability and Proliferation Assay

Cell viability was determined using MTT assay (MTT, Sigma), as defined earlier by Alam et al., 2011. Briefly, 20 ml of MTT (5 mg/ml) was added to each well containing a density of 1 × 10^4^ cells [parental OSCC cells, HPSE1-inhibited (shRNA HPSE1-) and -upregulated (orfRNA HPSE1+) clones]. After 3 h of incubation at 37°C, the cell supernatants were discarded, the MTT crystals were dissolved with DMSO, and the absorbance was measured at 570 nm at 12, 24, and 48 h. The percentage of viability was defined as the relative absorbance of transfected versus non-transfected control cells. The experiment was performed three independent times in quintuplicates.

### Migration Assay

To analyze the movement of the cell toward the artificial space, a scratch was created in a monolayer confluent cell, the oral cancer cell lines, and their respective HPSE1-inhibited and -upregulated clones were plated at 5 × 10^4^ in 12-well plates. When the cell monolayer reached 90–95% confluence, a scratch was made with a tip of 200 µL (time 0). Following 24 and 48 h, the images were recorded by a camera coupled to the inverted microscope (Nikon, Eclipse TS100) using Motic Image Plus 2.0 software.

### Adhesion Assay

For the assay, 96-well plates were coated with 10 ug/cm^2^ of type I collagen (Corning, Cat # 354236) in 100 ul of PBS for 24 h at 4°C. A quantity of 200 ul of PBS was used to wash the wells each three times, followed by covering with 200 ul of 3% BSA in PBS for 2 h at 37°C. Control wells were coated with only 3% BSA solution. Then, 3 × 10^3^ cells were harvested and resuspended in 100 ul DMEM/F12 medium supplemented with 3% BSA, added to each well, and incubated at 37^o^C/5% CO_2_ for 1 h. After washing the non-adherent cells, the remaining cells were fixed with 4% paraformaldehyde for 15 min and stained with 1% toluidine blue in 1% borax solution. The absorbance was measured at 650 nm by using an Epoch plate reader (Biotek).

### Invasion Assay

Transwell (Corning, United States) assay was carried out to examine OSCC cell invasion. Briefly, the membranes were coated with 50 ul of gelatinous matrix Miogel (2.4 mg/ml) with type I collagen (0.8 mg/ml) (Corning, Cat # 354236) (Salo et al., 2015) and incubated for 12–16 h. Then, the cells were plated in 200 ul of DMEM/F12 medium without FBS (8 × 10^4^ cells per well). As chemo-attractive, 500 ul of complete DMEM/F12 medium was added to the bottom layer of the inserts inside the wells, so the plates were incubated at 37°C/5% CO2 for 72 h. The invasion profile was assessed by carefully removing the formed membrane with cells from the inside of the insert.

The invading cells on the lower side of the insert were fixed in 4% paraformaldehyde for 15 min and stained with 1% toluidine blue in 1% borax solution. Then the cells were eluted in a 1% SDS solution for 5 min. Dye absorbance was measured at 650 nm in the Epoch plate reader (Biotek).

### Tube Formation Assay

To verify the potential HPSE1 modulation in the capillary structure formation, the oral cancer cells, the lines, and their respective inhibited and upregulated clones were grown in a culture medium of 10% FBS. After 70% confluence, the cells were incubated with SFB 0% for more than 24 h, and the preconditioned medium from each cell was collected. A measure of 50 µl of gelatinous matrix Miogel (2.4 mg/ml) with type I collagen (0.8 mg/ml) (Corning, Cat # 354236) (Salo et al., 2015) was polymerized in 96-well plates. HUVECs were incubated without FBS in RPMI1640 medium for 24 h. The cells were suspended in RPMI-1640 and added to wells coated with Miogel at a density of 1 × 10^4^ cell medium plus 80% the preconditioned medium and incubated at 37°C for 48 h. During the experimental period, cell morphology of capillary structures was observed and photo-documented at 12 and 24 h. The formation of the tube was examined and photographed under an inverted microscope. The quantification of antiangiogenic activity was done using Motic Image Plus 2.0 software and calculated by measuring the length of the tubule wall perimeter of endothelial cells.

### Statistical Analysis

The expression of HPSE1 among different tissues was analyzed and compared using the Kruskal–Wallis test with Dunn’s post hoc test, and the receiver operating characteristic (ROC) curve with area under the curve (AUC) was applied to compare the discriminatory ability of the HPSE1 between normal and tumor tissues. A chi-squared test was used to analyze the association of HPSE1 expression and clinicopathological features. The Kaplan–Meier method was used to construct survival curves that were compared using log-rank tests. The Cox proportional hazard model was used for univariate and multivariate survival analysis. For the multivariate analysis, the stepwise method, including the clinicopathological parameters verified in the univariate analysis as potential predictor variables, was performed to characterize the independent survival factors of OSCC patients. In this method, variables are sequentially included, and those that become non-significant are then removed. The final model is built only with variables displaying a *p* value ≤ 0.05. These statistical analyses were performed using MedCalc® statistical software, version 19.5.3 (MedCalc Software Ltd., Ostend, Belgium). All *in vitro* assays were performed at least three times, and data were analyzed using one- or two-way analysis of variance with repeated measures (RM-ANOVA) with post hoc comparisons based on Tukey’s multiple comparisons test. These analyses were performed using GraphPad Prism software version 5.0. For all statistical analyses, the significance level was set at 5% (*p* ≤ 0.05).

## Results

### Heparanase 1 is Overexpressed in the Oral Squamous Cell Carcinoma Cell Lines and Tissues

**FIGURE 1:**
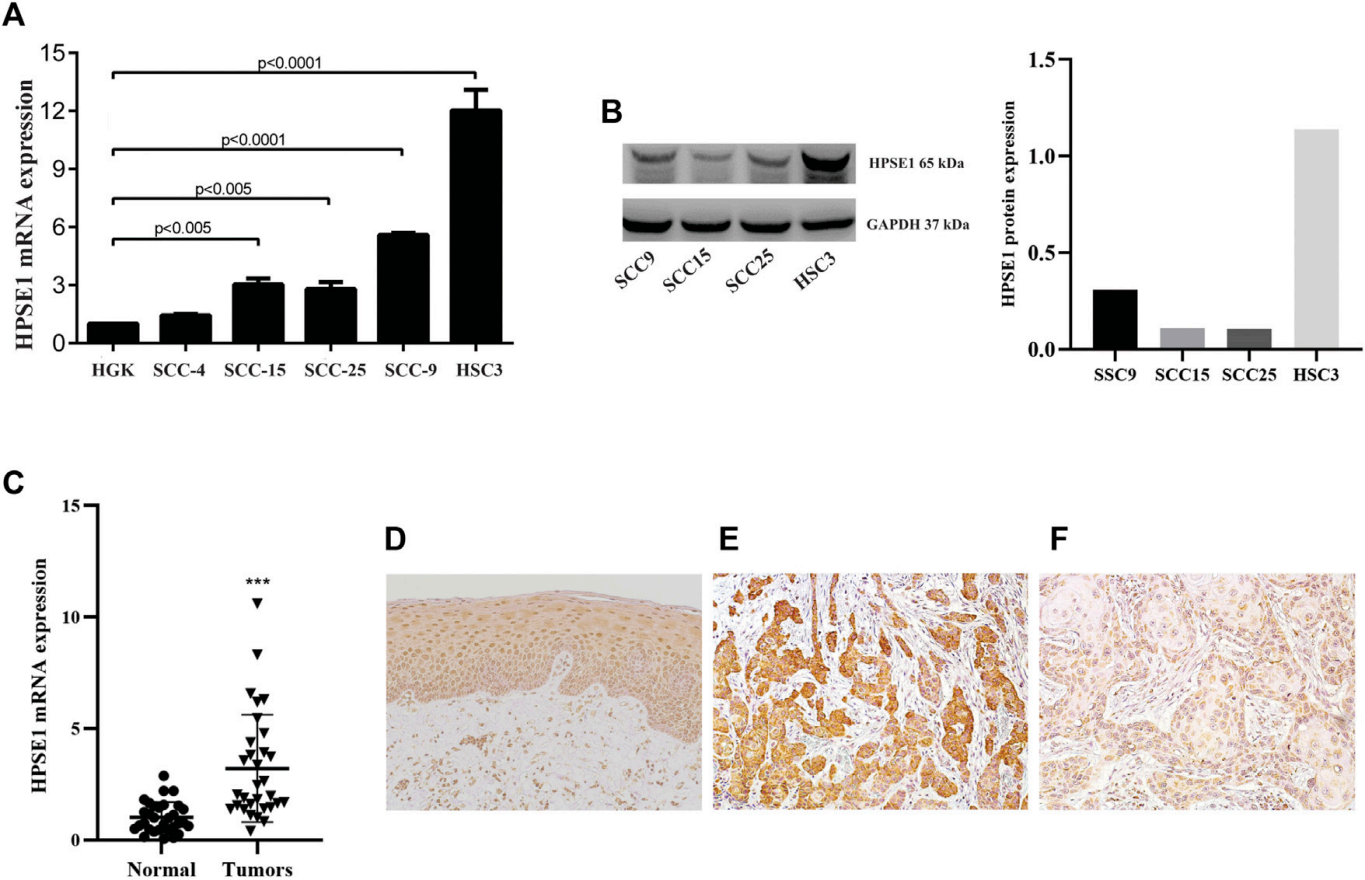
HPSE1 is overexpressed in OSCCs patient samples and OSCC-derived cell lines. Total RNA from fresh samples and cell lines was converted to cDNA and subjected to qPCR. For gene expression analysis of tissue samples relative quantification was based on the comparison of a pool of five normal oral tissues, while the spontaneously immortalized but non-transformed epithelial cell line HGK was used as reference for comparison with OSCC-derived cell lines. The levels of HPSE1 mRNA were significantly higher in OSCC cell lines compared with HGK cells **(A)**. The high expression levels of protein HPSE1 were confirmed on OSCC-derived cell lines by Western Blot analysis **(B)**. The levels of HPSE1 mRNA were also significantly higher in OSCC tissue samples compared to normal oral mucosa **(C)**. Representative images in a high-power field (200×) of immunohistochemical analysis for HPSE1 in Normal oral tissue **(D)** and OSCC tissue preparations confirmed its higher expression at protein level; with a distinct cytoplasmic distribution and intensity in oral cancer samples expressing both higher **(E)** and lower **(F)** levels of HPSE1. Results were statistically determined by ANOVA followed by Tukey multiple comparison test, where **p < 0.005, ***p < 0.001, and ****p < 0.0001.

The expression of HPSE1 was analyzed by qPCR, and the levels of HPSE1 mRNA were significantly higher in the OSCC cell lines SCC-15 (*p* < 0.005), SCC-25 (*p* < 0.005), SCC-9 (*p* < 0.001), and HSC-3 (*p* < 0.0001) compared with the immortalized, but not transformed, epithelial cell line HGK (Figure 1A). Western bloting analysis was carried to confirm HPSE1 expression in these OSCC cell lines (Figure 1B). The cell line SCC-9 was selected to carry out functional *in vitro* assays to investigate the HPSE1 role in oral cancer tumorigenesis. Of note, we checked the nuclear and cytoplasmic HPSE1 protein expression in the SCC-9 cell line; these OSCC cells showed higher HPSE1 expression in the cytoplasm compartment than in the non-malignant HGK cells, where the HPSE1 expression was more restricted to the nuclear fraction (Supplementary Figure S1A).

We also sought to determine HPSE1 mRNA expression levels in 35 fresh samples from OSCC and normal oral mucosa (*n* = 40). For that, equivalent concentrations of total RNA of these normal tissues were pooled and used as a reference for mRNA relative quantification. A significantly higher level of HPSE1 mRNA was observed in the group of tumors than the controls (*p* < 0.001, Figure 1B), and the variation on HPSE1 expression levels was very small among control samples. For instance, we also verified the HPSE1 expression pattern in a subset of 33 OSCCs, and in normal oral tissue (Figure 1D), by immunohistochemistry analysis. Interestingly, the immunostaining for tumor cells revealed that HPSE1 upregulation had marked staining intensity in the cytoplasm of OSCC tissues (Figures 1E,F). Immunoreactivity for HPSE1 was also detected in some inflammatory, endothelial, and stromal cells.

### Expression of Heparanase 1 is Related to Reduced Survival of Oral Squamous Cell Carcinoma Patients

**FIGURE 2:**
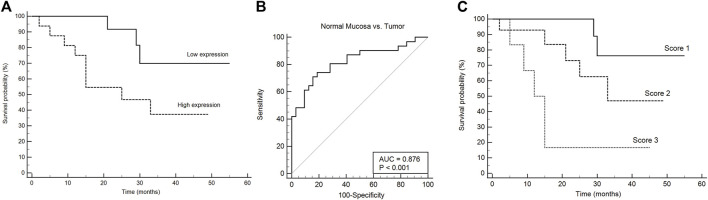
HPSE1 overexpression discriminates OSCC and normal samples and predicts low patient survival. **(A)** Kaplan–Meier cumulative curves for disease-free survival of patients with OSCC as a function of HPSE1 expression, showing a 70% probability of death in patients with higher expression of HPSE1 compared with those with low expression. **(B)** Receiver operating characteristic (ROC) curves showing the ability of the HPSE1 overexpression distinguishes OSCC tumors from normal oral samples. **(C)** Kaplan–Meier of multivariate survival analysis combining HPSE1 expression levels with pT stages, where score 1: HPSE1 lower expressing samples associated with pT1/2, score 2: HPSE1 lower expressing samples associated with pT3/4 or higher HPSE1 with pT1/2, and score 3: HPSE1 higher expressing samples associated with pT3/4. These results confirmed that HPSE1 overexpression was significantly associated with higher grade tumors.

We then assessed the association between mRNA expression of *HPSE1* and the clinical prognosis of OSCC patients. Patients with *HPSE1* overexpression had a significantly poorer disease-specific survival rate than those with lower expression. The probability of death was approximately 70% among patients expressing higher levels of *HPSE1*, significantly “higher” than the ones expressing lower *HPSE1* (37%) (*p* < 0.05, Figure 2A). The receiver operating characteristic (ROC) curve showed the ability of the *HPSE1* higher expression in discriminate OSCC tumors and normal oral samples (AUC = 0.876, *p* < 0.001) (Figure 2B). Also, recurrence was diagnosed in 30% of patients with higher expression of *HPSE1* after up to a 55-month follow-up, compared with 6.25% for those with low HPSE1 expression.

To investigate the association between mRNA expression levels of *HPSE1* and clinicopathological features, 35 OSCC patients were divided into *HPSE1* lower and higher expressing subgroups, with the median value as the cutoff. Considering the clinicopathological characteristics of tumors (Table 1), the overexpression of *HPSE1* in OSCC tissues was significantly associated with moderately and poorly differentiated histological grades of tumors (*p* = 0.02). Our results also demonstrate a significant correlation between the expression of *HPSE1* in tumor cells and cervical lymph node metastasis (pN stage), in that patients with metastasis (N+) had significantly higher HPSE1 expression (*p* = 0.02). Approximately 81% of the advanced stage tumors (pTNM stages III and IV) demonstrated high levels of *HPSE1*, while only 18% of pTNM stages I and II tumors presented high *HPSE1* expression (*p* = 0.04). Univariate analysis for disease-specific survival of patients with the OSCC cohort is also provided in Table 2. This analysis revealed that tumor size (pT3+pT4) [7.81 (95% CI 1.78–34.12, *p* = 0.006)], pTNM advanced stages (III and IV) [3.48 (95% 1.09–11.14, *p* = 0.035)], and barely lymphovascular invasion and HPSE1 expression were prognostic factors of the presented OSCC cohort. For HPSE1, a hazard ratio (HR) of 3.21 (95% CI 1.00–10.25, *p* = 0.047) was found for higher *HPSE1* mRNA expression about low *HPSE1* levels. Similarly, the presence of invasion in lymph vasculature had an HR of 3.88 (95% CI 1.02–14.82, *p* = 0.047).

**TABLE 2:** Univariate analysis for disease-specific survival of patients with oral squamous cell carcinoma.

	Overall survival	
	HR (95% CI)	*p* value
Age (years)		
< 61	1	0.54
≥ 61	1.42 (0.45–4.48)	
Gender		
Male	1	0.55
Female	0.64 (0.14–2.81)	
Smoking habit		
No	1	0.52
Yes	1.62 (0.36–7.24)	
Drinking habit		
No	1	0.24
Yes	2.73 (0.51–14.55)	
pT stage		
pT1 + pT2	1	^*^ **0.006**
pT3 + pT4	7.81 (1.78–34.12)	
pN stage		
N0	1	0.17
N+	2.23 (0.70–7.09)	
pTNM		
Early stage (I and II)	1	^*^ **0.035**
Advanced stage (III and IV)	3.48 (1.09–11.14)	
Location		
Tongue and floor of mouth	1	0.17
Other	2.34 (0.68–8.03)	
Tumor grading		
Well-differentiated	1	0.91
Moderately differentiated and poorly differentiated	0.93 (0.29–2.95)	
Treatment		
Surgery	1	^*^ **0.033**
Surgery + radiotherapy/chemotherapy	218.6 (1.50–31745.8)	
Margin status (mm)		
≥ 5	1	0.30
< 5	NA	
Perineural invasion		
No	1	0.17
Yes	2.24 (0.70–7.12)	
Lymphovascular invasion		
No	1	^*^ **0.047**
Yes	3.88 (1.02–14.82)	
Depth of invasion (mm)		
< 5	1	0.15
≥ 5	2.33 (0.72–7.49)	
Heparanase 1		
Low expression	1	^*^ **0.047**
High expression	3.21 (1.00–10.25)	

*p < 0.05. In bold, significant prognostic factors for OSCC cohort analyzed.

It was also observed that the size of the primary tumor (pT stage) was a risk factor for disease-specific survival [4.48 (95% CI 1.38–14.6, *p* = 0.0126)], but neither the local regional metastasis at diagnosis (pN stage) nor the expression of the HPSE1 mRNA itself (Supplementary Figure S2). To strengthen the prognostic data of the independent factors, we performed univariate and multivariate survival analyses by combining HPSE1 expression levels with the pT stage. For that, we categorized three different scores: score 1: HPSE1 lower expressing samples in association with pT1/2, score 2: HPSE1 lower expressing samples in association with pT3/4 or higher HPSE1 with pT1/2, and score 3: HPSE1 higher expressing samples in association with pT3/4. The multivariate Cox regression analyses of the combination of HPSE1 expression and tumor size (pT) discriminated well and confirmed that overexpression of this enzyme is significantly associated with more aggressive tumors of high rank [HR: 3.22 (95% CI: 1.42–7.32, *p* = 0.005)] (Figure 2C). Together, these findings suggest that the expression levels of HPSE1 could be used in association with the clinicopathological parameters to better predict the poor prognosis of OSCCs. Considering the closer *p*-value for HPSE1 expression as an independent factor for OSCC prognosis, future studies should consider increasing the patient sample cohort.

### Heparanase 1 Knockdown Increases Apoptosis and Suppresses the Proliferative Potentials of Oral Squamous Cell Carcinoma Cells

**FIGURE 3:**
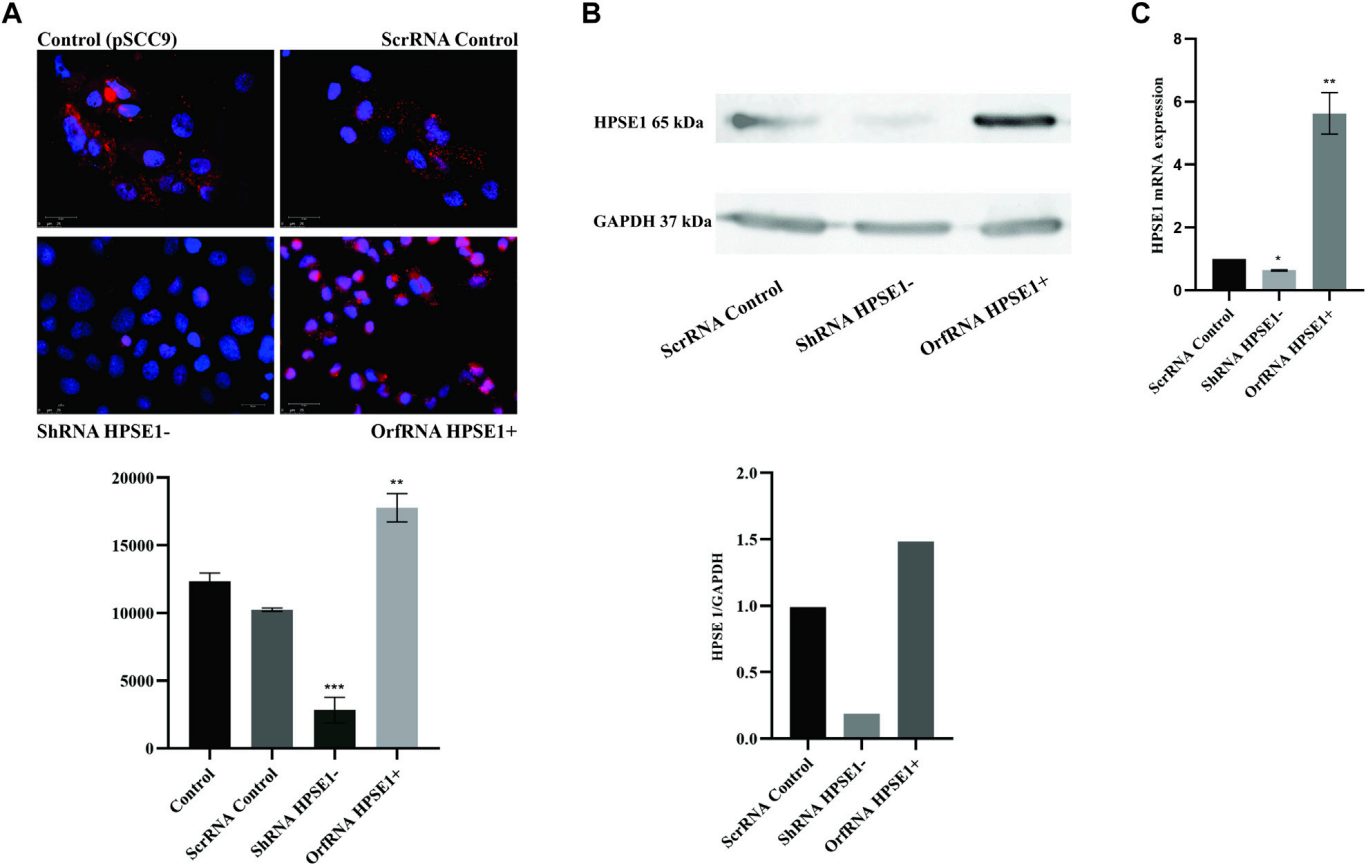
HPSE1 knockdown and upregulation efficiency in OSCC cells. The efficiency of endogenous HPSE1 modulation was verified by Immunofluorescence **(A)**, Western Blot **(B)** and RT-qPCR **(C)** analyzes. For the loss-of-function strategy, SCC-9 cells were transduced with shRNA expressing a vector sequence against HPSE1 (shRNA HPSE1−) and empty vector (scrRNA control). For the gain-of-function strategy, SCC-9 cells were transduced with a ORF clone in a shuttle vector to enhance HPSE1 expression (orfRNA HPSE1+), along with mock-transduced cells as described in *Methods*. shRNA HPSE1− cells showed a significant reduction and orfRNA HPSE+ cells showed a significant upregulation of HPSE1 in both mRNA and protein levels compared to scrRNA control and parental mock-transduced SCC-9 control cells (pSCC9), without targeting sequences. Results were statistically determined by ANOVA, followed by Tukey’s test, where *p < 0.05, **p < 0.01, ***p < 0.001, and ****p < 0.0001.

**FIGURE 4:**
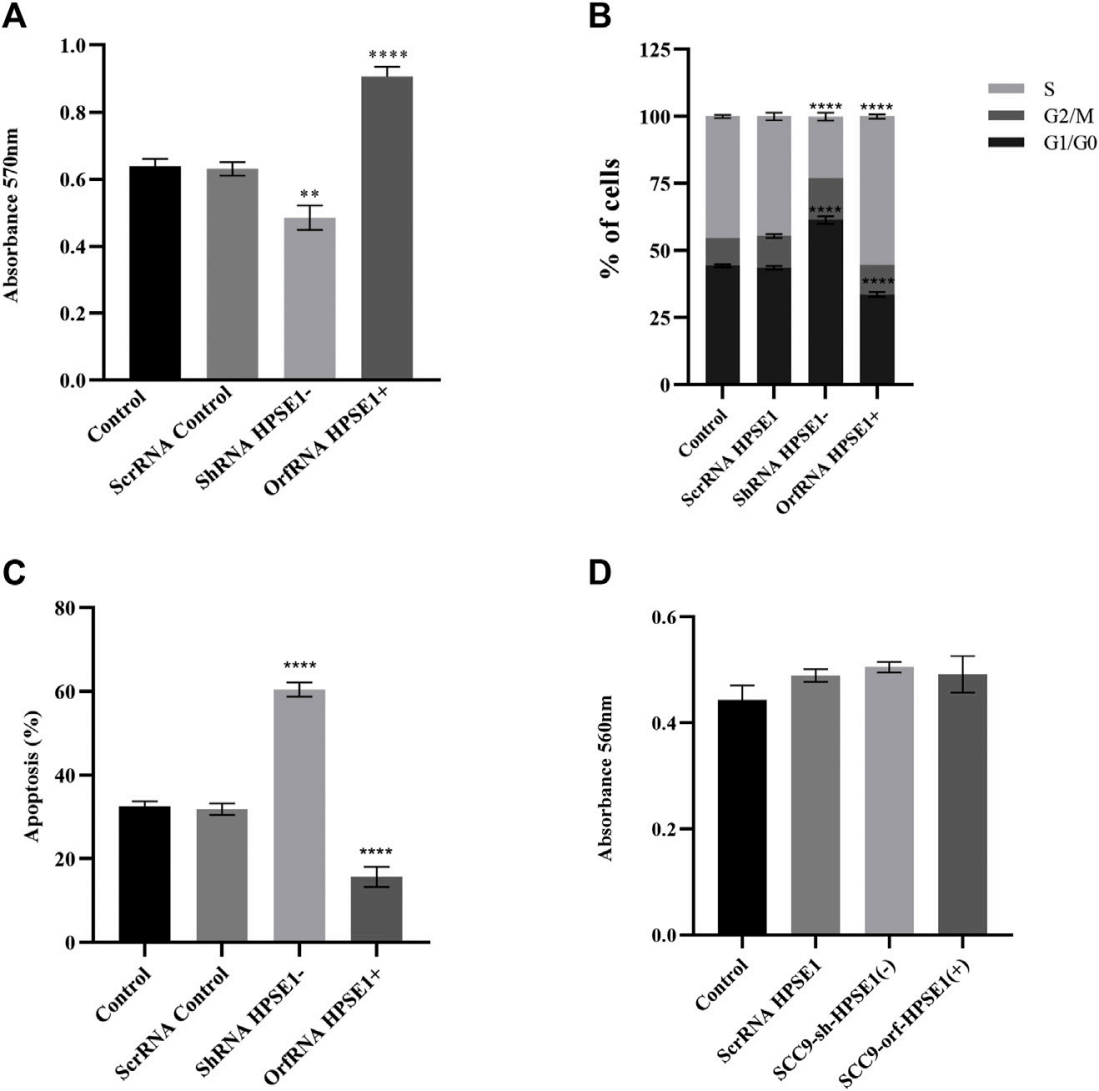
Downregulation of HPSE1 inhibits proliferation and enhances apoptosis of OSCC cells. Cells were subjected to MTT cell proliferation **(A)**, DNA content cell cycle analysis **(B)**, and apoptosis **(C)** assays. **(A)** Cell Proliferation Assay of the OSCC SCC9 cell line (Control) compared to empty vector SCC9 control clone (ScrRNA control), HPSE1 inhibitory/silencing SCC9-ShRNA HPSE1− clone, and the HPSE1 overexpressing SCC9-OrfRNA HPSE1+ clone. **(B)** Quantification of cell cycle analysis was performed by flow cytometry after staining with propidium iodide for the SCC-9 control cells and the respectives HPSE1-modulated clones. Abrogation of HPSE1 induced arrest of the SCC-9 cells cycle in G1 phase, and its upregulation significantly induced cell proliferation. **(C)** Flow cytometric analysis of apoptosis showed a remarkable increase in the number of apoptotic cells in HPSE1-silenced cells (ShRNA HPSE1−), while SCC-9 overexpressing HPSE1 (Orf-RNA HPSE1+) exhibited a reduction in apoptosis. **(D)** No significant differences in cell adhesion properties were observed among any transduced clones modulating HPSE1 expression, compared to parental mock-transduced cells (control). Plots compose experimental triplicate analysis and were statistically calculated using ANOVA followed by Tukey’s test, where *p < 0.05, **p < 0.01, ***p < 0.001, and ****p < 0.0001.

To gain insights into the role of HPSE1 in OSCC progression, HPSE1-silenced and -upregulated cells were achieved from the SCC-9 cell line by lentivirus-mediated shRNA and human GFP-tagged ORF plasmid, respectively. Conventionally, cells transduced with lentivirus carrying an shRNA-targeted sequence to HPSE1 exhibited a significant reduction in both HPSE1 protein (*p* < 0.001, Figures 3A,B) and mRNA (*p* < 0.05, Figure 3C) levels in comparison to parental SCC-9 cells (control) or cells transduced with the vector control (scrRNA), non-targeting sequence. Accordingly, cells transduced with the plasmid carrying a specific ORF sequence to enhance HPSE1 expression demonstrated a significant upregulation in both HPSE1 proteins (*p* < 0.01, Figures 4A,B) and mRNA (*p* < 0.01, Figure 3C) levels in comparison with parental and mock-transduced control cells. Of note, the cellular fraction analysis of the SCC-9 nuclear and cytoplasm compartments by WB analysis revealed that abrogation of HPSE1 protein expression was markedly reduced in the cytoplasmic fraction of shHPSE1-silenced cells and that ORF-mediated upregulation of HPSE1 enhanced cytoplasmatic expression of HPSE1 (Supplementary Figures 1B,C).

**FIGURE 5:**
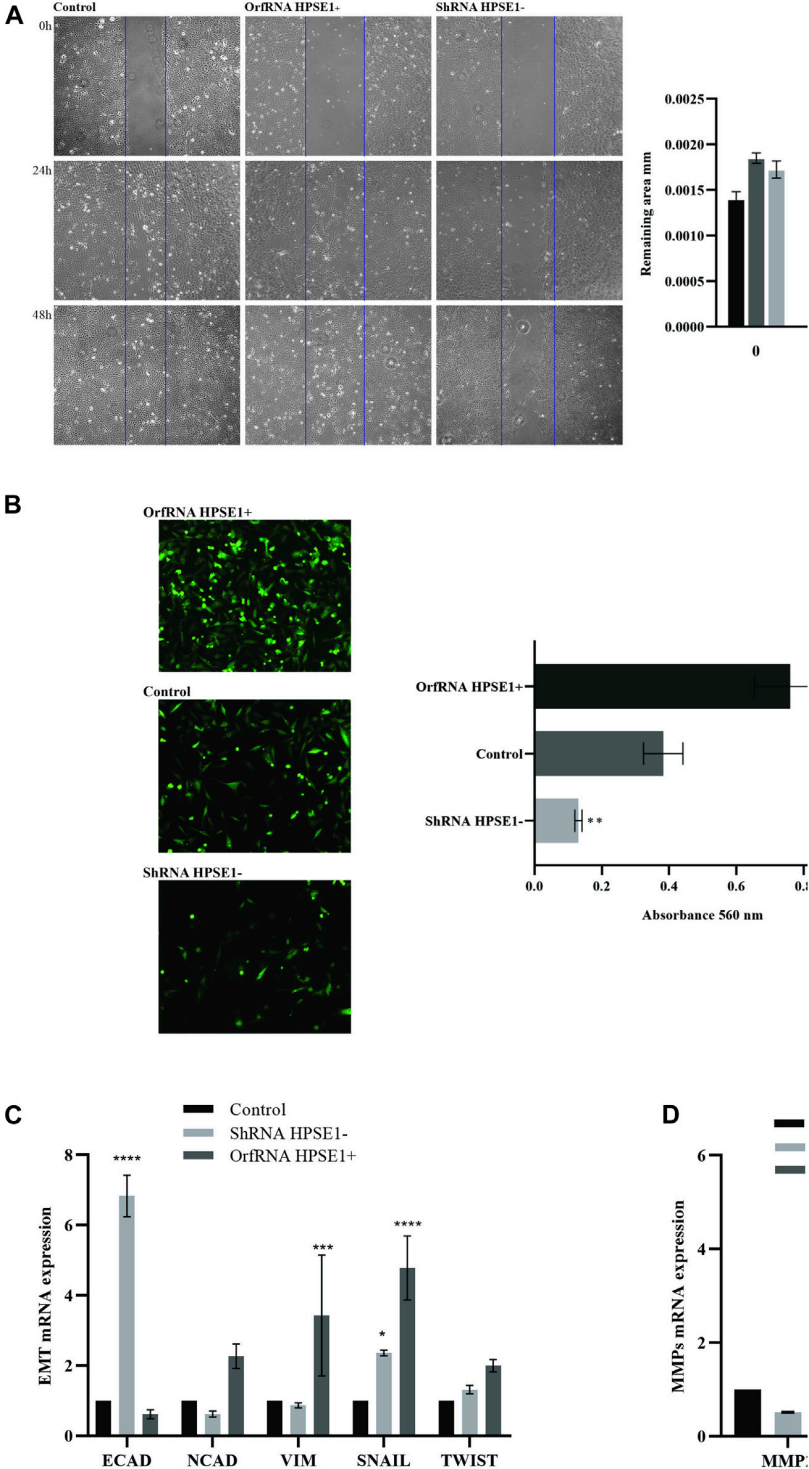
Overexpression of HPSE1 is associated with migration, invasion, ECM remodeling, and acquisition of EMT properties. **(A)** Photomicrographs of cell lines were taken 0, 24, and 48 h after wounding (40X). The average width of the lacunae was measured. Migration of SCC-9 cells was significantly decreased in HPSE1-silenced cells (ShRNA HPSE1−) and increased in HPSE1-upregulated clones (OrfRNA HPSE1+). Migration analysis based on this assay showed that cells with lower expression of HPSE1 closed the scratch wound significantly slower than the SCC9 Control cells. **(B)** Invasion of SCC-9 cells was significantly inhibited by HPSE1 knockdown, and significantly enhanced after its upregulation. **(C)** Analysing EMT markers, the downregulation of HPSE1 significantly induced the expression of E-Cadherin (E-CAD), while OrfRNA HPSE1+ SCC9 clones overexpressing heparanase had a significant increasing of Vimentin (VIM) and SNAIL expressions. **(D)** The upregulation of HPSE1 significantly enhanced the expressions of MMP2 and MMP9. All the graphs compile experimental triplicate analyses, and the results were obtained by ANOVA followed by Tukey assay, where *p < 0.05, **p < 0.01, ***p < 0.001, and ****p < 0.0001.

The specific shRNA against *HPSE1* drastically impaired the *in vitro* proliferation of SCC-9 cells (*p* < 0.01, Figure 5A). Accordingly, HPSE1 downregulation stimulates a significant increase in the cell amount at G0–G1 phases (*p* < 0.001) and an important reduction at the S phase (*p* < 0.0001) as compared to controls (Figure 4C). Conversely, orfHPSE1-upregulated cells showed enhancement in proliferation (*p* < 0.0001, Figure 4A), with marked reduction of cells at G0-G1 phases (*p* < 0.001) and an increase at the S phase (*p* < 0.0001, Figure 4C), compared to control cells. Also, HPSE1 upregulation was able to substantially decrease the number of cells in apoptosis (*p* < 0.0001, Figure 4B). Contrariwise, the number of apoptotic cells was significantly increased in SCC-9 shHPSE1-transfected cells (*p* < 0.0001, Figure 4B).

### Heparanase 1 Overexpression Is Associated With Migration, Invasion, Extracellular Matrix Remodeling, and Acquisition of Epithelial–Mesenchymal Transition Properties of Oral Squamous Cell Carcinoma Cells

During the process of invasion and metastasis, the epithelial aspect of tumor cells is lost as the mesenchymal phenotype is evoked, which characterizes the process known as EMT. To acquire an understanding of the role of HPSE1 in the regulation of EMT and invasion of oral cancer, we examined several features such as motility, invasiveness, adhesive capacity, expression of the epithelial marker E-cadherin, mesenchymal markers N-cadherin and vimentin, and ECM remodeling markers. The adhesive properties of HPSE1 in SCC9 cells were first investigated, and neither upregulation nor downregulation of HPSE1 affected SCC9 cell adhesion on surfaces coated with type 1 collagen (Figure 4D). Next, the scratch wound migration assay showed that upregulation of HPSE1 significantly enhanced the migration (*p* < 0.001, Figure 5A) and invasiveness (*p* < 0.001, Figure 5B) of SCC9-orfHPSE1 cells, whereas shHPSE1-silenced cells revealed significantly lower migration and invasion than the controls.

To further characterize the effects of HPSE1 on the expression of EMT and ECM remodeling makers, we examined the modulation of mRNA levels of *E-cadherin*, *vimentin*, *N-cadherin*, *Snail*, *Twist*, *MMP9*, and *MMP2* in HPS1-downregulated and -upregulated SCC9 cells by qRT-PCR. Significantly reduced levels of E-cadherin mRNA were observed in HPSE1-upregulated SCC9 cells (*p* < 0.05, Figure 5C). Concomitantly, HPSE1 increased the mRNA expression of the mesenchymal marker vimentin (*p* < 0.05), but not N-cadherin, and augmented the metalloproteinases MMP2 (*p* < 0.01) and MMP9 (*p* < 0.0001) (Figure 5D). Conversely, shHPSE1-silenced cells had upregulation of E-cadherin mRNA levels (*p* < 0.01), whereas diminishing expression of N-cadherin and vimentin was observed in these ShRNA SCC-9-transfected cells (Figure 5C) but not significatively compared to control cells. The mRNA expression of Snail, but not Twist, was higher in both down- and upregulated HPSE1-transfected SCC-9 cells, although it was much higher in the cells overexpressing HPSE1 (Figure 5C).

### Downregulation of Heparanase 1 Abrogates Endothelium Tube Formation and Induces Vascular Endothelial Growth Factor A Expression

**FIGURE 6:**
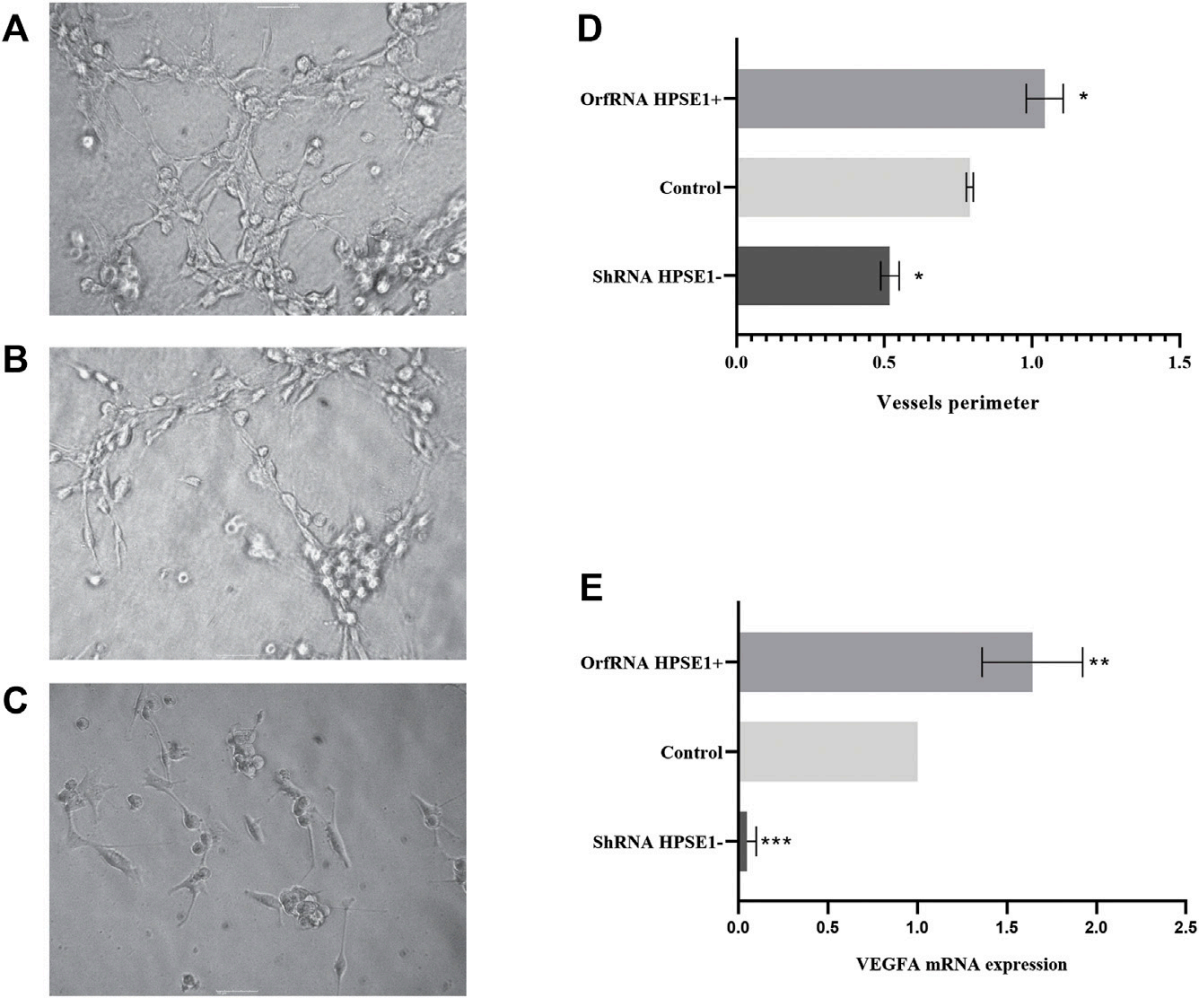
Overexpression of HPSE1 enhances tumor neo-vascularization and induces VEGFA expression. HUVEC cells on a Miogel 2D layer co-culture. Photomicrograph of HUVEC endothelial cells added with preconditioned medium from OSCC cell lines and their respective clones after 12 h of experiment: **(A)** HUVEC cells incubated with preconditioned medium of SCC9-OrfRNA HPSE1+ clone overexpressing HPSE1 (100×); **(B)** HUVEC cells incubated with preconditioned medium of parental SCC9 (Control) (100×); **(C)** HUVEC cells incubated with preconditioned medium of SCC9-ShRNA HPSE1- clone with reduction of HPSE1 expression (100×). **(D)** Quantification of measures of the vessel circumference perimeter for each one of the conditions; the graph compiles two experimental triplicate analysis. **(E)** Gene expression analysis of endothelial growth factor VEGFA by qRT-PCR; the graph compiles folded expression values of relative quantification by ddCT obtained through comparison of clones OrfRNA HPSE1+ and ShRNA HPSE1− relative to the control SCC9 cells (normal reference = 1). Normalization of the analysis was performed using the endogenous control gene, PPIA. Statistical tests were performed using ANOVA followed by Tukey’s test, where *p < 0.05, **p < 0.01, ***p < 0.001, and ****p < 0.0001.

We next assembled an *in vitro* tube formation assay, using human umbilical vein endothelial cell (HUVEC), to directly assess the possible effects of HPSE1 on oral cancer angiogenesis. HUVECs composed interlaced tubes in Miogel (Salo et al., 2015) (Figure 6). HPSE1-depleted cells demonstrated a significantly lower number of tubes than control cells (*p* < 0.05, Figures 6A–D). Conversely, HPSE1 upregulation drastically promoted HUVEC tube formation (*p* < 0.05, Figures 6A–D). Afterward, we assessed whether endogenous HPSE1 modulation could interfere with the expression of the vascular endothelial growth factor A (VEGFA) gene, often associated with angiogenesis, vasculogenesis, and endothelial cell growth induction. As shown in Figure 6E, significantly reduced levels of VEGFA mRNA were observed in HPSE1-silenced SCC9 cells (*p* < 0.001), whereas markedly higher expression of VEGFA was detected in HPSE1-upregulated cells (*p* < 0.01) than the controls.

## Discussion

Although predictive factors such as age, clinical stage, and histological grade have been used to assess prognosis (Rodrigues et al., 2014; Almangush et al., 2015; Sawazaki-Calone et al., 2015), they still have poor predictive power. In addition to that, none of the currently molecular markers have yet been able to provide adequately knowledge to benefit these patients (Søland and Brusevold, 2013). Therefore, it is essential to identify biomarkers that can accurately identify and stratify low- and high-risk oral cancer patients, improving the capacity to predict those who would benefit from a more complex and intense treatment. The mammalian endoglycosidase HPSE1 is the predominant enzyme responsible for degrading heparan sulfate activity and is known to coordinate multiple biological activities to promote tumor growth, invasion, metastasis, angiogenesis, and inflammation (Vlodavsky et al., 2018). HPSE1 accomplishes this by cleaving heparan sulfate and thereby regulating the bioavailability of heparin-binding proteins, stimulating the tumor microenvironment (TME), resolving the tumor–host crosstalk, and inducing the activation of various genes, signaling pathways, and the assembly of exosomes and autophagy (Vlodavsky et al., 2018). Taken together, these events ultimately influence tumor cell performance and their role in chemoresistance.

HPSE1 expression has also been previously shown to be elevated in several cancer types, including OSCC (Kurokawa et al., 2003; Väyrynen et al., 2018), and the overexpression was an indicator of worse prognosis (Rivara et al., 2016; Sanderson et al., 2017). So, in the present study, we sought to explore the potential roles of HPSE1 in OSCC biology. First, we confirmed that while heparanase mRNA and protein levels were expressed at very low levels in normal tissues, its expression was increased four times in primary tumors and was associated with poor prognosis, thus depicting the clinical relevance of HPSE1 in OSCC and positioning this enzyme as a valid drug target. Also, we verify that the probability of death was significantly higher among patients expressing higher levels of HPSE1 than the ones expressing lower levels. These results were in line with Vlodavsky et al. (2012) who also found that HPSE1 expression was associated with OSCC tumor invasion patterns in a xenograft model. Furthermore, high HPSE1 levels in primary tumor samples were significantly correlated with expanded malignity and the presence of metastatic disease (Hunter et al., 2014). These data corroborate with the suggestion that the upregulation of HPSE1 in primary tumors might increase its tendency to metastasize locally and remain remarkably upregulated in these tumor cells, which might as well successfully conquer distant organs (Vlodavsky et al., 2012).

Further analysis demonstrated that shortened disease-specific survival of OSCC patients was significantly associated with increased HPSE1 expression. In the univariate analyses, overexpression of HPSE1 proved to be a predictor of disease-specific survival along with consistent prognostic factors for OSCCs, such as tumors with high T classification, presence of cervical lymph node metastasis (N stage), and lymphovascular invasion. The multivariate Cox regression analyses of the combination of HPSE1 expression and tumor size (pT) confirmed that overexpression of this enzyme is significantly associated with higher grade more aggressive tumors. High levels of HPSE1 were also significantly associated with poorer prognosis, confirming HPSE1 as a prognosticator tool for OSCC. Indeed, recent studies reported the correlation of heparanase expression levels with a higher degree of the tumor, late stage, advanced tumor angiogenesis, and low patient survival (Jin and Zhou, 2017). Also, it is strongly suggested that dysregulated gene expression, a key feature of cancer, drives the overexpression of HPSE in the tumor microenvironment (TME), leading to pathological remodeling of the ECM and the release of cancer-promoting HSBPs (Hanahan and Weinberg, 2011; Hammond et al., 2014). HPSE also exhibits a variety of non-enzymatic functions, such as regulating gene expression, promoting cell adhesion, and tumor-promoting procoagulant activity (Nadir and Brenner, 2014; Sanderson et al., 2017). Thus, overexpression of HPSE in cancers promotes tumor growth and metastasis, leading to poor clinical prognosis (Rivara et al., 2016).

In the present study, we also showed that the localization of HPSE1 in oral cancer cells, from patients and cell lines, seemed to be correlated with the aggressiveness potential of those cells. Our results of immunohistochemical staining for heparanase showed marked positivity in oral cancer cells, particularly in the cytoplasmic compartment. Following these results, Leiser et al. (2011) reported that cytoplasmic localization of HPSE1 was associated with high-grade oral carcinomas.

Also, there have been pieces of evidence that the cytoplasmatic HPSE1 regulates the secretion, composition, and behavior of tumor cell-derived exosomes (Thompson et al., 2013). Other studies indicated that HPSE1 activates the syndecan–syntenin–ALIX pathway forming a linear axis that rules exosome formation (Roucourt et al., 2015; Rangarajan et al., 2020). Interestingly, ALIX-mediated syndecans and syntenin have been implicated in restraining exosome biogenesis, and HPSE1 was found to stimulate the secretion of exosomes and modify their composition and biological function (Vlodavsky et al., 2018). That scenario explains the relevant role of heparanase because once released, tumor-derived exosomes can travel through the body and impact resident cells at locations distal to the tumor, thereby aiding in the preparation of the pre-metastatic niche and influencing metastatic organotropism.

HPSE1 was also overexpressed in different OSCC-derived cell lines (up to 15-fold higher), and it seemed to be proportionally more highly expressed according to the malignant potential of these cell lines. We then modulated endogenous HPSE1 by generating stable OSCC cell lines expressing shRNA and ORF RNA-targeting HPSE1 to better understand the part of HPSE1 in the preclinical stage. We demonstrated that the abrogation of HPSE1 on oral cancer cells increases apoptosis, while suppressing proliferation, migration, invasion, epithelial–mesenchymal transition (EMT), and angiogenesis. In agreement with other studies, HPSE1 knockdown had expressive unfavorable effects on the viability and proliferative potential of OSCC cells (Ikuta et al., 2001; Tatsumi et al., 2020) as overexpression of HPSE1 increased the proliferation of OSCC cells. Per previous studies (Ikuta et al., 2001), we also demonstrated that HPSE1 regulates migration and invasion status of OSCC cells, which may explain why higher HPSE1 expression was significantly correlated with a worse prognosis.

In other cancers, HPSE1 activation has been linked to metastasis and tumor progression upholding. Chen et al. (2015) suggested an association between heparanase expression and cell adhesion, and metastasis in hepatocellular carcinoma cell lines. Previous studies (Tatsumi et al., 2020) also reported that HPSE1 played a role in the regulation of proliferation and autophagy in normal and malignant cells, conferring survival benefits and resistance to chemotherapy. We then explore the association of the proliferative process with HPSE1 expression in oral cancer biology. Our study confirmed that the increase in apoptosis resulted from HPSE1 suppression, as well as the inhibition of cell proliferation, while its overexpression reduced apoptosis and increased proliferation in these oral cancer cells. Furthermore, in the spontaneous bladder cancer mouse model, heparanase silencing significantly suppressed bladder cancer invasion. Our results showed that HPSE1 suppression significantly reduced the ability of cancer cells to migrate and invade, along with the modulation of the mRNA expression of EMT and ECM markers.

In this context, it is important to highlight that the TME is a cellular and a non-cellular environment that contains the tumor, and includes fibroblasts, surrounding blood vessels, bone marrow-derived immune cells, lymphocytes, signaling molecules, and an extracellular matrix. Also, the EMT involves a biological process by which cells transit between epithelial and mesenchymal states by decreasing cell–cell adhesion and increasing cell motility and metastasis. In cells with HPSE1 depletion, we observed an induction of E-cadherin and inhibition of vimentin, and the overexpression of HPSE1 promotes the EMT by upregulating these mesenchymal markers, an important phenotype for invasion and metastasis. The heparanase produced by cancer cells has also been suggested to cleave HS of the basement membrane (BM), which is necessary for them to infiltrate the BM of blood vessels during metastasis. In the ECM, HPSE1 can also discharge HS-bound growth factors and proteolytic enzymes, which promotes angiogenesis and proliferation of cancer cells, further assisting invasion and metastasis (Jayatilleke and Hulett, 2020). Traditionally, the ECM protein network, glycoproteins, and proteoglycans were noticed as an embedded scaffold furnishing a structural framework for cells to form tissue organs. Later, it was observed that the ECM played a role in the management of cell survival, proliferation, and differentiation. The enzymatic activity of HPSE leads to ECM remodeling by increasing bioavailability and function of heparan sulfate (HS)-binding proteins (HSBPs) (Cohen-Kaplan et al., 2008; Knelson et al., 2014), which are important components of the ECM due to their contribution to the maintenance of ECM structural integrity and regulatory functions (Sarrazin et al., 2011). In corroboration with our findings, high HPSE1 expression induced head and neck cancer progression (Cohen-Kaplan et al., 2008) and mammary adenocarcinoma cell invasion and metastasis correlated with loss of E-cadherin and gain of MMPs 2 and 9 levels (Welch et al., 1990).

Finally, as HPSE1 overexpression has been shown to strengthen tumor angiogenesis in other contexts, Vlodavsky et al. (2018) investigated the effects of HPSE1 silencing and overexpression on *in vitro* endothelial tube formation. As shown in the present study, the preconditioned medium overexpressing HPSE1 significantly enhanced and the HSPE1-inhibited preconditioned medium significantly decreased vascular tube formation compared to the preconditioned medium with HPSE1 wild-type (WT) expressing cells. Interestingly the formation of tubes structures of HUVEC cells treated with the preconditioned medium of HPSE1-overexpressing clones persisted, in contrast to those treated with preconditioned medium from the parental SCC9 control cells. When treated with the inhibition clone-preconditioned medium, there was a complete vessel breakdown after 24 h, suggesting a relevant role of HPSE1 in the maintenance of blood capillary vessels in the process of tumor angiogenesis. Thus, our data support the hypothesis that the level of HPSE1 produced by cancer cells can facilitate angiogenesis, which in turn supports tumor growth.

Angiogenesis, the formation of new blood vessels, has physiologic and pathological roles (Chung et al., 2010). To better understand the HPSE1 role in molecular pathways associated with angiogenesis, we evaluated the VEGFA gene expression profile in OSCC cell lines with modulated HPSE1 expression. Our results showed that VEGFA expression was increased in OSCC cell lines overexpressing HPSE1 and decreased in HPSE1-silenced OSCC cells when compared to OSCC cell line parental control. The VEGF-signaling pathway is a major regulator of tumor growth and metastasis (Vlodavsky et al., 2018). Heparanase aids tumor invasion by shattering the ECM, releasing angiogenic factors, and recruiting an angiogenic environment. Heparanase activity has been well implicated in cell dissemination associated with the processes of inflammation and angiogenesis (Parish et al., 2013), through the release of a combination of growth factors linked to bFGF, VEGF, and HB-EGF e KGF, which support neovascularization and wound healing. Zcharia et al. (2005) reported that high levels of heparanase in wound healing accelerated tissue repair and skin restoration, mediated mainly by an increased angiogenic response. These actions make heparanase a promising target for cancer therapy (Vlodavsky et al., 2018). Monoclonal antibodies against VEGFA were the first angiogenesis inhibitors authorized for the treatment of colon cancer patients with metastasis based on their survival benefit (Hurwitz et al., 2004) and could also be a tool to combat advanced oral cancer disease.

Of note, most local and distant metastatic cases had higher expression of HPSE1, while most non-metastatic cancers showed lower HPSE1 expression. That may be analogous to the HPSE1 role in various aspects of cancer metastasis (i.e., while in some cancer phases, heparanase assists ECM degradation; in others, it enhances neovascularization). Nevertheless, it is very reasonable that an outstanding correlation endures between heparanase expression and activity and the metastatic potential of oral cancer cells.

This report was the first to simultaneously investigate heparanase modulation (inhibition and induction) and its effects on the suppression of cancer migration, invasion, proliferation, apoptosis, and angiogenesis of oral cancer. However, our data have limitation on the correlation between HPSE1 and the EMT markers and VEGFA in clinical OSCC tissue specimens due to lack of any more samples available to perform such analyses. Thus, as a preclinical-based design, particularly in number of samples, this study has its limitation, and to gain more detailed insights into the molecular crisis involving the HPSE1-mediated OSCC progression, additional *in vivo* mechanistic studies are required.

## Conclusion

Taken together, our results suggest that HPSE1 is overexpressed in patients with OSCC and OSCC-derived cell lines, upregulation of HPSE1 is significantly associated with poor outcome, and HPSE1 implicates an important role in OSCC migration, invasiveness, and the angiogenic process. On this basis, further studies should be carried on to establish HPSE1 as a routine prognostic marker for patients with oral cavity cancer.

## Data Availability

The original contributions presented in the study are included in the article/Supplementary Material, further inquiries can be directed to the corresponding author.

## Supplementary Material

Supplementary Figure S1 Analysis of Immunofluorescence (IF) and Western Blot (WB) from OSCC cellular compartments. **(A)** In the SCC-9 cell line, nuclear and cytoplasmic HPSE1 protein expression was determined by IF and showed higher HPSE1 expression in the cytoplasmic compartment compared to the non-malignant HGK cells, in which HPSE1 expression was more restricted to the nuclear fraction. **(B)** Western blotting showing the distribution of HPSE1 protein expression pattern and its quantification **(C)** in SCC9 cells, and the respective suppressed (ShRNA) and upregulated (OrfRNA) derived clones within the subcellular compartments. The results showed a significant reduction in cytoplasmic protein levels of OSCC by HPSE1-shRNA silencing, while its induced upregulation significantly increased cytoplasmic HPSE1 expression in SCC-9 cells. N, Nucleus; C, Citoplasm.

Supplementary Figure S2 Multivariate analyses showed a significant association of combinations of HPSE1 levels and pTNM **(A)**, pT **(B)**, and pN **(C)** stages for Disease Free-Survival. Survival curves indicated largely improved discriminatory ability.
