# Supplementary material for: Heparanase 1 Upregulation Promotes Tumor Progression and Is a Predictor of Low Survival for Oral Cancer
Source: Front Cell Dev Biol. 2022 Jun 16;10:742213. doi: 10.3389/fcell.2022.742213 (PMC9629395; doi:10.3389/fcell.2022.742213)
Supplement: Supplementary file 1 [file Table1.docx]

**Supplementary Table 1.** Summary description of clinical-epidemiopathological data from samples of all thirty-five individuals with OSCC included in this study.

| **Age (years)** | **N (%)** |
| --- | --- |
| < 62 years | 16 (45.7) |
| ≥ 62 years | 19 (54.3) |
| **Gender** |  |
| Male | 27 (77.2) |
| Female | 8 (22.8) |
| **Smoking habit** |  |
| No | 3 (8.6) |
| Yes | 32 (91.4) |
| **Drinking habit** |  |
| No | 8 (22.8) |
| Yes | 27 (77.2) |
| **pT stages*** |  |
| pT1 | 10 (28.6) |
| pT2 | 16 (45.7) |
| pT3 | 7 (20.0) |
| pT4 | 2 (5.7) |
| **pN stages*** |  |
| N0 | 23 (65.7) |
| N+ | 12 (34.3) |
| **Location** |  |
| Tongue | 15 (42.9) |
| Floor of mouth | 9 (25.7) |
| Labial | 5 (14.3) |
| Buccal mucosa | 3 (8.6) |
| Retromolar | 2 (5.7) |
| Palate | 1 (2.8) |
| **Histological grade**** |  |
| Well-differentiated | 20 (57.1) |
| Moderately-differentiated | 12 (34.3) |
| Poorly-differentiated | 3 (8.6) |
| **Treatment** |  |
| Surgery | 26 (74.3) |
| Surgery + Radiotherapy | 7 (20.0) |
| Surgery + Radiotherapy and Chemotherapy | 2 (5.7) |

* Acoording to the International Union Against Cancer (TNM stage); **World Health Organization (WHO).
