# Supplementary material for: Heparanase 1 Upregulation Promotes Tumor Progression and Is a Predictor of Low Survival for Oral Cancer
Source: Front Cell Dev Biol. 2022 Jun 16;10:742213. doi: 10.3389/fcell.2022.742213 (PMC9629395; doi:10.3389/fcell.2022.742213)
Supplement: Supplementary file 4 [file Table2.docx]

**Supplementary Table 2.** Sequence of primers for gene expression analysis by qRT-PCR.

| **Primers** | **Sequence** |
| --- | --- |
| HPSE1 Foward | 5' TGGCAAGAAGGTCTGGTTAGGAGA 3' |
| HPSE1 Reverse | 5' GCAAAGGTGTCGGATAGCAAGGG 3' |
| PPIA Foward | 5’ GCTTTGGGTCCAGGAATGG 3’ |
| PPIA Reverse | 5’GTTGTCCACAGTCAGCAATGGT 3’ |
| MMP2 Foward | 5'TGATCTTGACCAGAATACCATCGA3' |
| MMP2 Reverse | 5'GGCTTGCGAGGGAAGAAGTT3' |
| MMP9 Foward | 5'TGGGGGGCAACTCGGC3' |
| MMP9 Reverse | 5'GGAATGATCTAAGCCCAG3' |
| N-CADHERIN Foward | 5’TGGGAATCCGACGAATGG3’ |
| N-CADHERIN Reverse | 5’CGTACGGCGCTGGGTATC3’ |
| E-CADHERIN Foward | 5’ACAGCCCCGCCTTATGATT3’ |
| E-CADHERIN Reverse | 5’ TCGGAACCGCTTCCTTCA 3’ |
| VIMENTIN Foward | 5’ GGCTCGTCACCTTCGTGAAT 3’ |
| VIMENTIN Reverse | 5’ TCAATGTCAAGGGCCATCTTAA 3’ |
| SNAIL Foward | 5’ GCGTGTGCTCGGACCTTCT 3’ |
| SNAIL Reverse | 5’ ATCCTGAGCAGCCGGACTCT 3’ |
| TWIST Foward | 5’ GCGCTGCGGAAGATCATC 3’ |
| TWIST Reverse | 5’ GCTTGAGGGTCTGAATCTTGCT 3’ |
| VEGFA Foward | 5' CGAGGGCCTGGAGTGTGT 3' |
| VEGFA Reverse | 5' CGCATAATCTGCATGGTGATG 3' |
